# Analysis of NDNF and SLC1A2 gene expression in the dorsolateral prefrontal cortex of individuals with autism

**DOI:** 10.3389/fnins.2026.1694827

**Published:** 2026-02-16

**Authors:** Ariel Moraes de Andrade, Larysy Raquelly Vidal de Souza, Rodrigo Freire Oliveira, Karina Maia Paiva, Felipe Porto Fiuza, Pedro Henrique Silva de Farias, Roque Ribeiro da Silva Júnior, Maria Vanessa Freitas Holanda, Thales Allyrio Araújo de Medeiros Fernandes, Paulo Leonardo Araújo de Góis Morais, José Rodolfo Lopes de Paiva Cavalcanti

**Affiliations:** 1 Laboratory of Experimental Neurology, Department of Biomedical Health, State University of Rio Grande do Norte, Mossoró, Brazil; 2 Ana Bezerra University Hospital (HUAB/EBSERH/UFRN), Santa Cruz, RN, Brazil; 3 Postgraduate Program in Neuroengineering, Edmond and Lily Safra International Institute of Neurosciences, Santos Dumont Institute, Macaíba, Brazil; 4 Laboratory of Biochemistry and Molecular Biology, Department of Biomedical Health, State University of Rio Grande do Norte, Mossoró, Brazil

**Keywords:** Autism Spectrum Disorder, dorsolateral prefrontal cortex, gene expression, NDNF, SLC1A2 (EAAT2)

## Abstract

**Introduction:**

This study investigated the expression of NDNF and SLC1A2 in the dorsolateral prefrontal cortex of individuals with Autism Spectrum Disorder (ASD), a region linked to executive functions, emotional regulation, social skills, and sensory processing.

**Methods:**

Using data from the Allen Human Brain Atlas – Autism Study, 17 post-mortem cases (ASD: 9; controls: 8; ages 2–14) were analyzed with *in situ* hybridization and Nissl staining. Histological images were processed with FIJI-ImageJ and Ilastik software.

**Results:**

No significant differences in NDNF expression were observed, though a trend toward lower levels in ASD was noted. In contrast, SLC1A2 expression was significantly increased in ASD and showed age-related growth, possibly reflecting neuroinflammatory processes. Nissl staining indicated similar neuronal density between groups, suggesting that gene expression changes may reflect functional alterations rather than cell number.

**Conclusion:**

These results highlight complex neurogenesis alterations in ASD and underscore the need for further research to identify biomarkers and potential therapeutic targets.

## Introduction

Autism Spectrum Disorder (ASD) is a neurodevelopmental condition with both genetic and environmental origins. According to the DSM-5 TR, diagnosis requires social impairments alongside restricted and repetitive behaviors, with manifestations varying by developmental stage and age (American Psychiatric Association [APA], 2023). Altered functioning of specific brain regions is central to the autism phenotype (Courchesne et al., 2011).

Atypical development occurs in several regions, including the prefrontal and temporal cortex, amygdala, hippocampus, cerebellum, and striatum, affecting neuroanatomical features and synaptic connectivity, particularly in dendritic shafts and spines (Lamanna and Meldolesi, 2024). Children with ASD exhibit increased neuron numbers in the dorsolateral prefrontal cortex (DLPFC), a region critical for behavioral control, attention, working memory, decision-making, creativity, and emotional regulation (Courchesne et al., 2011; Seminowicz and Moayedi, 2017). Functional gradients along the rostro-caudal axis of the frontal cortex show hierarchical organization, with caudal areas linked to concrete functions and rostral regions to abstract cognitive processes (Amiez and Petrides, 2018; Bahlmann et al., 2014).

Although early childhood is marked by brain overgrowth in ASD, adulthood is characterized by cortical thinning and neuronal loss, indicating that adult studies may miss developmental abnormalities present in children (Stoner et al., 2014). Excessive prenatal neuron and synapse formation may underlie early overgrowth, contributing to ASD clinical features. Additionally, altered protein and gene expression in the prefrontal cortex suggests synaptic aberrations and neurotransmitter dysregulation (Chow et al., 2012). Dysregulation of genes involved in DNA repair, cell cycle, apoptosis, differentiation, and neural organization may drive abnormal brain growth and cytoarchitecture.

This study investigates the excitatory–inhibitory synapse balance in the prefrontal cortex of individuals with ASD using VGlut1 (presynaptic) and PSD-95 (postsynaptic). Layer II of BA9 and BA47 exhibited increased excitatory synapses and reduced inhibitory synapses across layers, leading to cortical hyperexcitability and disrupted cognitive networks (Vakilzadeh et al., 2024). Post-mortem analyses of selective molecular markers in autistic and neurotypical children further support these findings, highlighting alterations in excitatory/inhibitory neurons and glial cells, alongside candidate autism-related genes (Stoner et al., 2014; Holanda et al., 2025).

Among the genes related to neurodevelopment and synaptic regulation, NDNF (Neuron-Derived Neurotrophic Factor) and SLC1A2 (Excitatory Amino Acid Transporter 2) stand out, particularly for the investigation of ASD (Autism Spectrum Disorder). Its expression throughout development suggests that subtle alterations may contribute to atypical cortical organization and connectivity dysfunctions.

The neuron-derived neurotrophic factor is a gene that exhibits robust, consistent, and specific expression patterns in the cortex of individuals with autism and is expressed exclusively in neurons. Its expression has also been reported in multipotent mesenchymal stromal cells from the neocortex, umbilical cord blood, and bone marrow (Kuang et al., 2010; Zhang et al., 2019). Furthermore, it has several biological functions related to restorative processes, including the promotion of cell growth and migration and the inhibition of apoptosis.

The SLC1A2 gene, on the other hand, codes for the excitatory amino acid transporter EAAT2, which is responsible for regulating extracellular glutamate in the cerebral cortex, being essential for glutamatergic balance and the prevention of excitotoxicity. Considering evidence of glutamatergic dysregulation and cortical hyperexcitability in ASD, alterations in SLC1A2 expression may reflect compensatory or adaptive mechanisms associated with synaptic dysfunction, neuroinflammation, and age-dependent molecular changes.

Studies using glutamate transporter knockouts have shown that loss of the EAAT2 protein following antisense treatment leads to elevated extracellular glutamate levels, neurotoxicity observed at both light and electron microscopy levels, and neurodegeneration characteristic of excitotoxicity (Rothstein et al., 1996).

Thus, evidence suggests that ASD is associated with an imbalance between excitatory and inhibitory signaling, often described as hyperexcitability, related to neuronal organization and dysfunctions in cortical circuits. Modulations in the expression of these genes during critical periods of development may impact the formation, stabilization, and functioning of neural networks, influencing the excitatory–inhibitory dynamics observed in ASD (Stoner et al., 2014; Holanda et al., 2025).

Given that ASD is characterized by alterations that begin during development, post-mortem analyses across different ages remain fundamental for identifying persistent or adaptive molecular signatures over time. Accordingly, the analysis of gene expression in the dorsolateral prefrontal cortex in this study allows the investigation of alterations reflected in detectable molecular patterns, contributing to the understanding of atypical cortical organization.

Our objective was to describe the expression of NDNF, which promotes neurogenesis, cell growth, migration, and inhibits apoptosis, and SLC1A2, encoding the EAAT2 glutamate transporter linked to neuronal toxicity. We quantified neurons expressing these genes in the dorsolateral prefrontal cortex, comparing them to total cell counts via Nissl staining, aiming to identify potential diagnostic differences (Qu et al., 2022).

## Methods

### Samples

The gene analysis was conducted using histological images from the Allen Human Brain Atlas – Autism Study, developed by the Allen Institute for Brain Science in collaboration with the University of California. The database provides high-resolution post-mortem human brain samples from donors supplied by the National Institute of Child Health and Human Development Brain and Tissue Bank for Developmental Disorders, the Brain and Tissue Bank for Developmental Disorders, and the Harvard Brain Tissue Resource Center, under agreements permitting academic research use.

A total of 42 freshly frozen cortical tissue blocks (1–2 cm^3^) from the superior or middle frontal gyrus of the dorsolateral prefrontal cortex, posterior superior temporal cortex, or occipital cortex were obtained from autistic and control donors aged 2–14 years. RNA quality was assessed using high-resolution electrophoretic trace analysis with the Agilent Bioanalyzer 2100.

The study focused on high-resolution images of the dorsolateral prefrontal cortex from 22 donors (11 ASD, 11 controls; 8 males and 3 females per group). Six cases were excluded due to missing NDNF or SLC1A2 expression data, resulting in 17 male donors aged 2–14 years. All donor information was anonymized to ensure confidentiality. Regarding the information related to sample localization, the cerebral hemisphere of origin was not consistently available for all samples. Only samples numbered 706112, 706114, and 706116 had documented localization as originating from the left hemisphere dorsolateral prefrontal cortex. Therefore, hemisphere was not used as an inclusion criterion and is considered a limitation in the interpretation of the results, particularly in the context of potential functional asymmetries.

The causes of death were varied, and no specific information regarding the cause of death was made available. The postmortem interval and RNA quality were previously evaluated and controlled by the Allen Institute for Brain Science, ensuring that the samples were suitable for histological and gene expression analyses. Additional detailed clinical information, such as comorbidities or medication use, was not systematically available due to the anonymous and secondary nature of the database.

The analysis included 25 high-risk genes and cell markers. Gene classification followed three criteria: (1) reduced or absent expression compared to controls, (2) abnormal expression based on the number of labeled cells relative to area, and (3) cell-type or layer-specific expression abnormalities (Stoner et al., 2014). Among the selected genes were NDNF (Neurotrophic Factor Derived from Neurons) and SLC1A2, which encodes the Excitatory Amino Acid Transporter 2 (EAAT2, or GLT-1).

Tissue samples were processed using *in situ* hybridization (ISH) to localize specific RNA sequences. Frozen samples were sectioned with a Leica CM3050 S cryostat and labeled using a digoxigenin-based staining method. Nissl staining was also performed to visualize neuronal cells; slides were stored at 37 °C for 1–5 days and removed 5–15 min before staining, with dye highlighting key neuronal features (Allen Institute for Brain Science, 2013).

For the study, no special permissions, authorizations, or protocols were required to use the material in research and publications, provided it was not for commercial purposes and adhered to the Citation Policy^1^ and the Institute’s Code of Ethics^2^.

### Histological procedures

The following descriptions are available in the technical report titled “*IN SITU* HYBRIDIZATION IN THE ALLEN HUMAN BRAIN ATLAS.”^3^ The tissue samples for this study were provided by the following brain tissue banks: National Institute of Child Health and Human Development Brain and Tissue Bank for Developmental Disorders (Baltimore, MD) through contracts N01-HD-4-3368 and N01-HD-4-3383, as well as the Brain and Tissue Bank for Developmental Disorders (Miami, FL), the Autism Tissue Program (Princeton, NJ), and the Harvard Brain Tissue Resource Center (Belmont, MA) (Allen Institute for Brain Science, 2013).

### Image extraction

The database provides brain tissue samples from donors through links that direct users to high-resolution images of both *in situ* hybridization and Nissl staining for the selected gene. It also includes donor and sample information, along with the option to download the images. Additionally, the cell locations are identified by their position in the dataset and can be adjusted using a micrometer (μm) scale bar.

**FIGURE 1:**
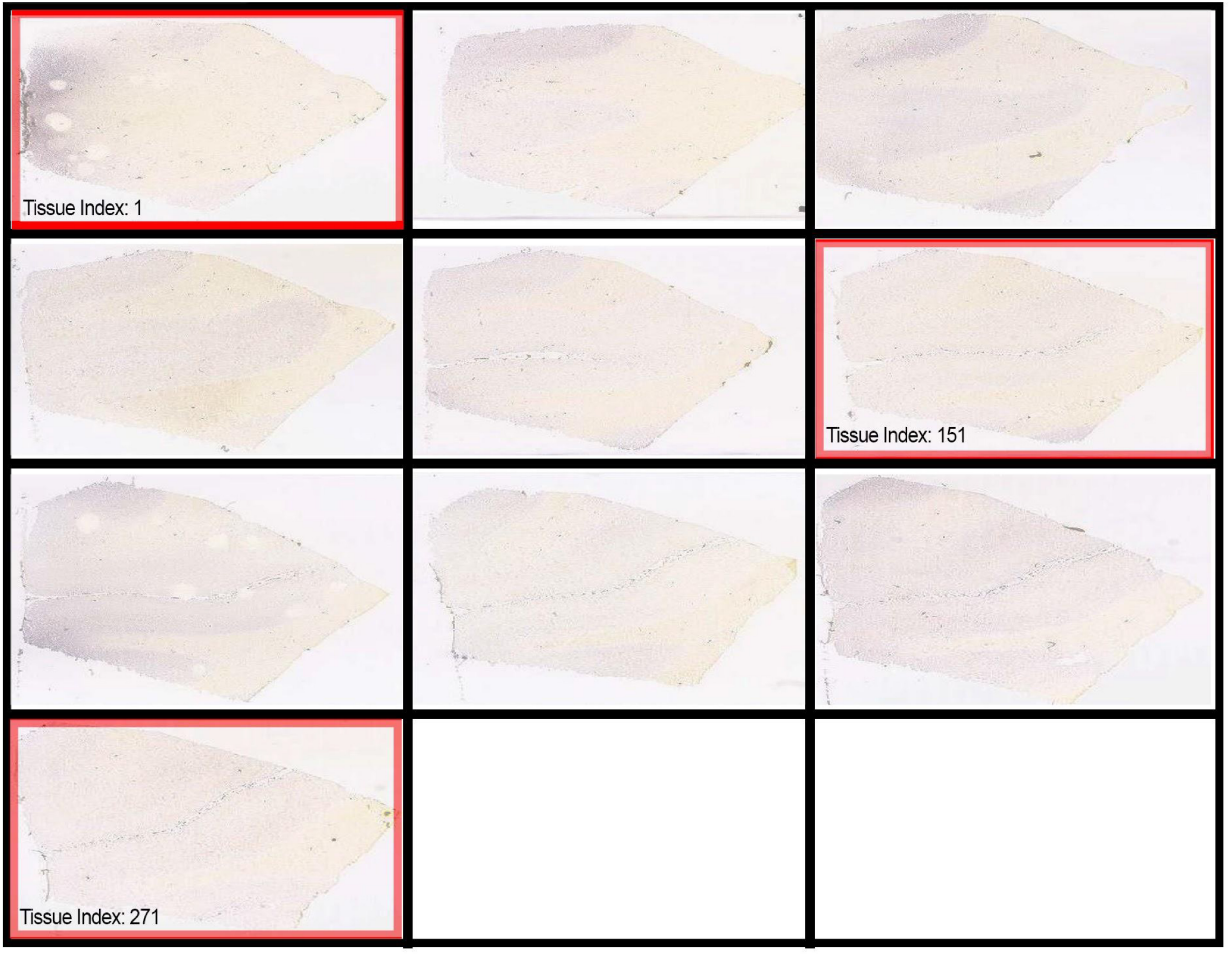
Identification of sections used for analysis. The red rectangles indicate the selection of images from case B6399 in ISH. (Credit for web page and histological images: Allen Institute, within the Allen Human Brain Atlas database) Available at: https://human.brain-map.org/ish/experiment/show/100034529.

For this study, images were selected for the SL1CA2 gene, a specific marker for the excitatory amino acid transporter, and NDNF, a marker for the neurotrophic factor derived from neurons. Specifically, three sections were chosen, corresponding to: section 1 (first image), section 2 (fifth image), section 3 (tenth image), as indicated in the Figure 1.

**FIGURE 2:**
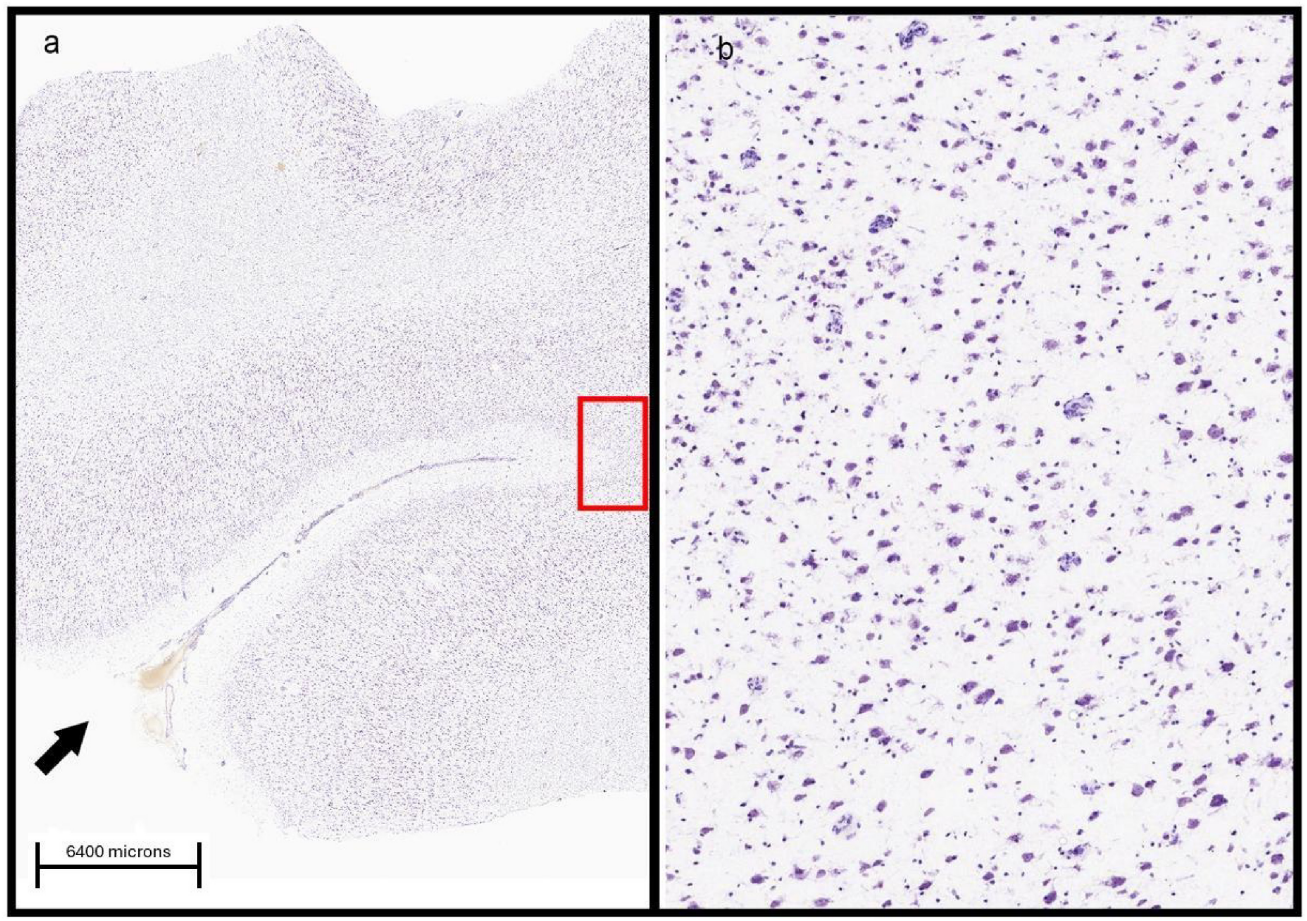
Example of standardization for sectioning. The arrow indicates the sulcus to guide the cut, and the red rectangle indicates the sectioning area for case UMB4898 (NDNF) in Nissl staining **(a)**. In panel **(b)**, the area in panel **(a)** is magnified (sectioned). Credit for web page and histological images: Allen Institute. https://human.brain-map.org/ish/experiment/show/100032527.

After extracting the images of interest from the database, a strategy was developed to segment the tissue area to quantify neurons and protein expression. The image cropping was performed using the open-source software FIJI-ImageJ, version 1.52p. The cropping format was based on the study Layer-Specific Changes in the Prefrontal Glia/Neuron Ratio Characterizes Patches of Gene Expression Disorganization in Children with Autism (Rabelo et al., 2023). Additionally, Nissl-stained images were overlaid with ISH images to ensure the segmentation was performed in corresponding areas, as indicated in the Figure 2.

Two main quantitative measures were performed: The average number of cells expressing the gene of interest (detected by ISH); the average total number of cells (detected by Nissl staining). Additionally, cell density was calculated based on the relationship between gene expression, cell count, and area.

The measurements were obtained from three tissue sections. For each analyzed individual, the sum of values from all three sections was divided by three, ensuring a representative mean estimate of the sample. Furthermore, additional metrics were calculated to better interpret the distribution and proportion of gene expression and cells in the analyzed tissues. The cell density calculation considered the number of gene-expressing cells relative to the total number of cells, allowing for an evaluation of the relative concentration of expressing cells within the investigated area. This metric followed a similar principle to Neubauer chamber cell counting, where cell density is determined by the ratio between the number of cells and the corresponding area.

The data underwent descriptive statistical analysis to determine normal distribution using the Shapiro-Wilk test. Given this: for non-parametric data found in NDNF, the Mann-Whitney test was used. For parametric data found in SLC1A1, the Unpaired *t*-test was applied. In addition, Pearson correlations were computed to assess associations with age-related variables. The results of this analysis provide insights into whether significant differences exist in the mean and cellular density of gene expression between ASD and control groups, offering valuable information on potential alterations associated with ASD.

## Results

In the analysis of the average number of NDNF-labeled cells, assessed using the Shapiro-Wilk test (*W* = 0.7212; *p* = 0.0063 for ASD and *W* = 0.7835; *p* = 0.0280 for the control group), no significant difference was found between the ASD and control groups (Mann-Whitney U = 22; *p* = 0.8048). Upon analyzing the graphs, a substantial overlap between the groups was observed, although the ASD group showed a lower median value. Nevertheless, no statistically significant differences in NDNF expression were found between the ASD and control groups.

**FIGURE 3:**
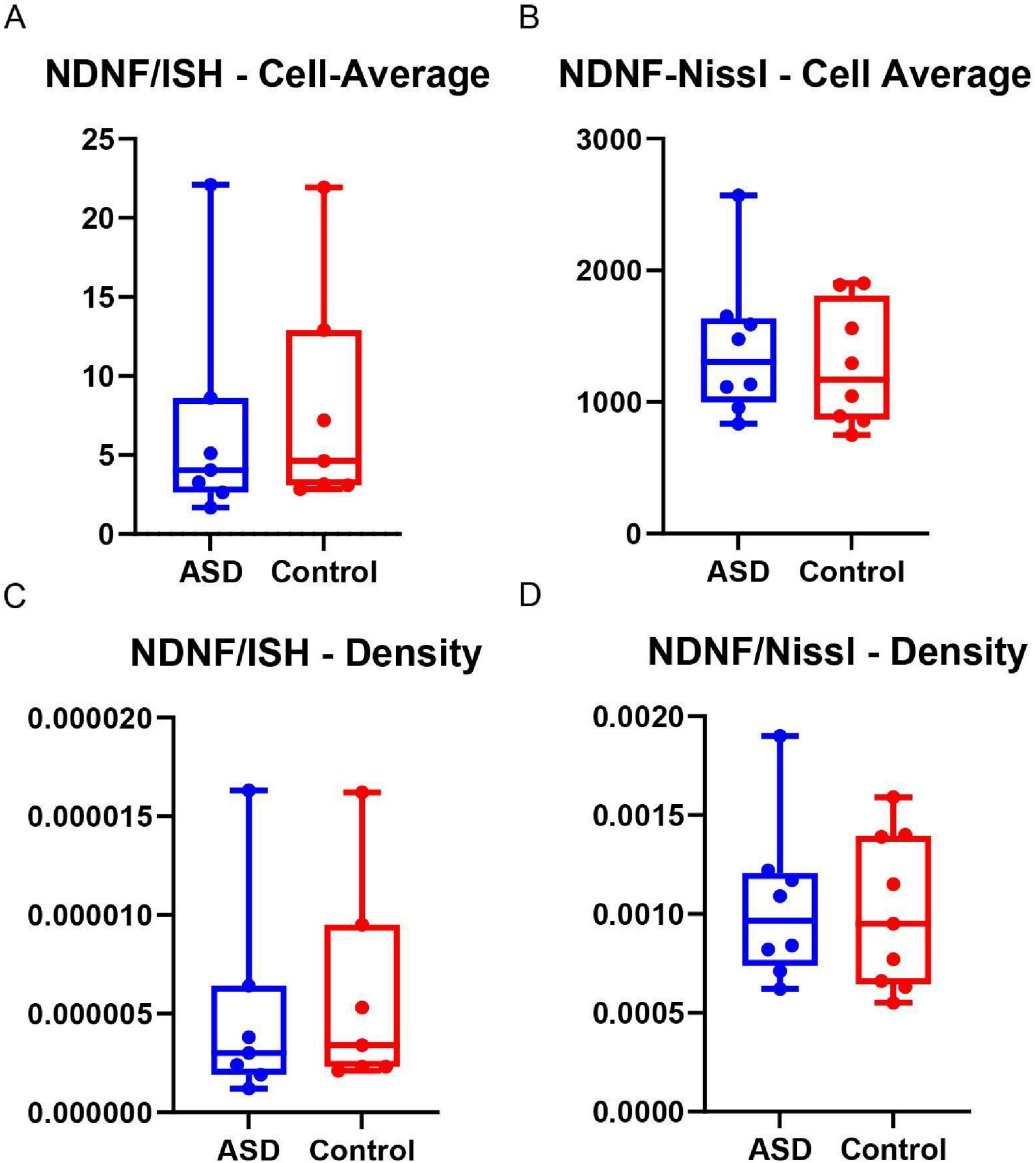
Graphs **(A,B)** present the comparison of the average number of cells between the ASD and control groups. **(A)** Shows cells indicating NDNF expression identified through ISH, and **(B)** demonstrates the cells present in the total sample, indicated by Nissl staining. No significant statistical differences were identified between the groups evaluated by the average number of cells **(A,B)** and by density **(C,D)**. However, the graphs indicate a trend of lower expression in the ASD group. No asterisks are shown, as no comparisons reached statistical significance (*p* > 0.05).

The NDNF density data followed the same pattern as the average and ratio, showing no statistical difference (Mann-Whitney U = 22; *p* = 0.7786). The graphical representation also indicates a decrease in NDNF in the ASD group, but without statistical significance, as indicated in the Figure 3.

For the average number of Nissl-stained cells, no statistically significant difference was identified (*t* = 0.5571; df = 14; *p* = 0.5862) between the ASD and control groups. Similarly, the density analysis showed no statistically significant difference (*t* = 0.1882; df = 15; *p* = 0.8532).

**FIGURE 4:**
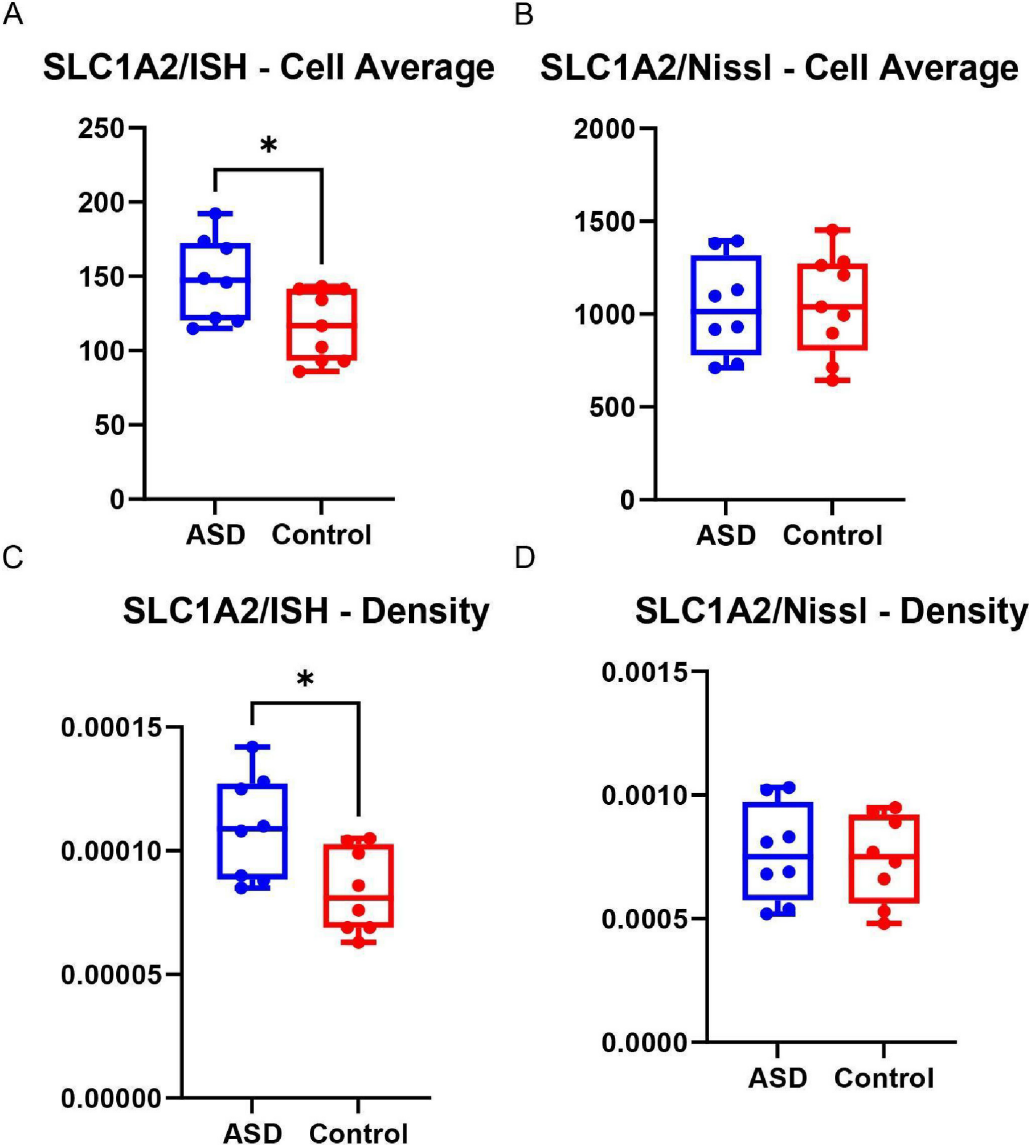
Graphs **(A,B)** demonstrate the comparison of the average number of cells between the ASD and control groups, where **(A)** shows cells expressing SLC1A2 through ISH, and **(B)** shows the cells present in the sample. Graphs **(C,D)** show the comparison of cellular density in the ASD and control groups, with **(C)** representing the density of cells expressing SLC1A2, and **(D)** showing the cells found via Nissl. The “*” indicates statistically significant differences (*p* < 0.05) in graphs **(A,C)**, reflecting increased SLC1A2 expression and cellular density in the ASD group compared to controls. Were observed in graphs **(A,C)**, with an increase in the number of cells expressing SLC1A2 in the ASD group compared to the control group when assessed through ISH.

In the analysis of the average number of SLC1A2-labeled cells, a statistically significant difference was observed (*t* = 2.482; df = 15; *p* = 0.0254), suggesting an increase in the average number of SLC1A2-labeled cells in the ASD group compared to the control group, as indicated in the graph, as indicated in the Figure 4.

The analysis of the average cellular density labeled by SLC1A2/ISH between the ASD and control groups also indicated a statistically significant difference (*t* = 2.683; df = 14; *p* = 0.0178). Box plot graphs show that the ASD group exhibited higher cellular density compared to the control group.

Additionally, cellular density values in Nissl-stained tissue and the average number of cells in both ASD and control groups did not show a statistically significant difference (*p* = 0.8128; *t* = 0.2413), indicating similar expression levels of this variable between the analyzed groups.

Regarding age and NDNF expression, the results of the Pearson correlation test between the donors’ age (ASD and control groups) and the average cell count and cellular density for the NDNF and SLC1A2 genes were obtained. The data indicate that the average cell count for NDNF showed a moderate correlation (*r* = 0.5231), but without statistical significance (*p* = 0.1834). The cellular density for NDNF showed a correlation of *r* = −0.2871, also without statistical significance (*p* = 0.4905).

For the SLC1A2 gene, the average cell count showed a weak correlation with age (*r* = −0.2542) and no statistical significance (*p* = 0.5434). However, the cellular density for SLC1A2 revealed a strong correlation (*r* = 0.7277), which was statistically significant (*p* = 0.0407), suggesting a relevant association between the density of cells expressing the gene and the age of the donors. The results indicate that the older the age, the higher the cellular density in the ASD group compared to the control group.

## Discussion

The literature does not extensively explore the role of NDNF in ASD, but it is known that this gene plays crucial roles in neurogenesis and the maintenance of neuronal plasticity. A study conducted on the brains of mice during developmental periods, using western blot and immunohistochemistry techniques, found that NDNF is expressed in the brain and spinal cord. It was also observed that NDNF was expressed by various types of neurons, including Cajal-Retzius cells, cortical neurons, hippocampal neurons, olfactory mitral cells, Purkinje cells, and spinal neurons. However, its expression decreased significantly from the seventh postnatal day to adulthood, suggesting its importance in neurogenesis (Kuang et al., 2010).

The analysis of NDNF did not reveal statistically significant differences between the groups. Although the research indicates the presence of the gene and the graphs do show visible differences, where there are fewer cells expressing NDNF in ASD individuals compared to control individuals, these findings should be interpreted with caution and do not allow conclusions regarding the relevance of NDNF gene alterations in the context of ASD. Future studies with larger sample sizes and a cell-type–specific approach may help determine whether subtle expression differences exist.

Results related to the gene SLC1A2 showed a significant increase in both the average number of cells expressing the gene and cellular density in the ASD group. The literature associates this gene with the regulation of glutamate transport, which is crucial for synaptic homeostasis (Malik and Willnow, 2019). This increase may represent an attempt to mitigate synaptic excitotoxicity and neural toxicity, which are commonly observed in individuals with ASD (Rothstein et al., 1996).

Alterations in SLC1A2 expression are also related to neurological conditions such as epilepsy, reinforcing the hypothesis that imbalances in glutamatergic signaling may be involved in neurodevelopmental disorders (Qu et al., 2022). It can also be suggested that SLC1A2 overexpression may indicate an attempt to compensate for high levels of extracellular glutamate, reducing the risks of synaptic dysfunction and neurodegeneration.

The relationship between SLC1A2 cellular density and the donors’ age also proved significant in the ASD group, indicating that changes in the expression of this gene may be more pronounced in older individuals. This result is consistent with the literature that identifies age-dependent gene expression changes in individuals with ASD (Chow et al., 2012).

The increased expression of SLC1A2 suggests the activation of compensatory mechanisms, possibly in response to glutamatergic signaling. Although the direct assessment of excitotoxicity markers was beyond the scope of this study, these findings provide a solid foundation for investigating how this modulation contributes to the preservation of synaptic homeostasis. The positive association observed suggests that factors such as chronic neuroinflammation and oxidative stress, both described as more evident in later stages of life, may influence SLC1A2 expression in ASD (Courchesne et al., 2011).

The analysis of Nissl staining data revealed no statistically significant differences for either NDNF or SLC1A2. This result reinforces the hypothesis that specific changes in genes may be more related to gene function than to total cellular quantity or density.

In collaboration with Stoner et al. (2014) in the study of patches, comparing stereological density measurements using adjacent Nissl sections to sections showing patches in the dorsolateral prefrontal cortex, no change in neuron count was found. These observations suggest that changes in the number of neurons may be regional or specific to cellular subtypes, as no statistical differences were observed in the present study. It is important to highlight that the findings refer to the total neuronal population content, without evaluating specific cell subtypes, which underscores the need for further studies to explore these differences in greater depth, in addition to the small number of subjects in the study.

This study presents some methodological limitations that should be taken into consideration, including the sample size, the exclusive inclusion of male donors, the lack of assessment of specific cellular subtypes, limited information on hemispheric laterality, and the absence of detailed clinical variables, which may affect a more refined interpretation of the findings.

The clinical heterogeneity among donors, which is not available for analysis, may contribute to interindividual variability, especially in genes sensitive to physiological or inflammatory states or to medication use. Despite these limitations, all samples were rigorously controlled and assessed for preserved RNA integrity, reducing the likelihood that the findings reflect methodological artifacts.

Nevertheless, this research provides relevant evidence regarding the expression of genes associated with ASD and highlights the need for future investigations to further elucidate the neurobiological mechanisms underlying ASD. It is also important to note that, although qRT-PCR is a well-established method for quantifying gene expression, it is not applicable within the context of this dataset, which consists exclusively of high-resolution histological images.

In contrast, quantification based on *in situ* hybridization (ISH) has been widely employed in post-mortem autism studies, as it enables the evaluation of laminar patterns and cell-density distributions–parameters that cannot be captured through bulk RNA quantification.

## Conclusion

From this perspective, this study identifies differential molecular patterns in the dorsolateral prefrontal cortex of individuals with ASD, characterized by increased SLC1A2 expression and unchanged NDNF levels. These findings suggest altered glutamatergic regulation without significant changes in overall neuronal density, supporting the hypothesis that functional molecular alterations, rather than gross cellular differences, contribute to ASD neuropathology. Together, these results reinforce the relevance of glutamate homeostasis in ASD and highlight the need for integrative developmental and molecular investigations to identify reliable biomarkers and therapeutic targets.

## Data Availability

The datasets presented in this study can be found in online repositories. The names of the repository/repositories and accession number(s) can be found in the article/supplementary material.

## Footnotes

1
https://alleninstitute.org/

2
https://alleninstitute.org/legal/terms-use/

3
http://help.brain-map.org/display/humanbrain/Documentation
